# Supplementary material for: The Anti-Glucocorticoid Receptor Antibody Clone 5E4: Raising Awareness of Unspecific Antibody Binding
Source: Int J Mol Sci. 2022 May 2;23(9):5049. doi: 10.3390/ijms23095049 (PMC9104323; doi:10.3390/ijms23095049)
Supplement: Supplementary file 1 [file ijms-23-05049-s001.zip › ijms-1681063-supplementary.pdf]

Supplementary Materials

Tables

Supplementary Table S1: Mass spectrometric analyses of top enriched proteins immunoprecipitated from whole cell lysates using the anti-GR (5E4) antibody

	Gene name	Unique peptides	MW [kDa]	Intensity	iBAQ	MS/MS count	fold change (log2)	p-value (-log10)
**HEK293**								
1	AMPD2	43	100,69	16639000000	415980000	308	13,443	3,436
2	TRIM28	37	88,549	9313400000	291040000	282	12,508	5,969
3	STIM1	20	77,422	1368800000	42776000	56	8,945	6,142
4	FSD1	17	55,819	624910000	24996000	49	7,397	1,819
5	NDUFA5	6	13,459	443870000	88774000	31	7,222	3,442
6	WAPAL	19	132,94	344040000	4986100	40	7,176	4,546
7	PRRC2C	25	308,77	460020000	4600200	94	7,162	4,126
8	NDUFS3	13	30,241	859670000	50569000	64	7,137	4,719
9	CKAP5	49	218,52	1061600000	10307000	157	6,715	2,810
10	NR3C1	16	82,903	254870000	6216400	41	6,316	4,137
**Jurkat**								
1	AMPD2	43	100,69	16639000000	415980000	308	14,472	6,380
2	STIM1	20	77,422	1368800000	42776000	56	9,470	4,967
3	AMPD3	3	78,492	51006000	2040300	4	7,246	3,315
4	NR3C1	16	82,903	254870000	6216400	41	7,068	4,294
5	CDK11	24	89,437	1090800000	26605000	125	6,836	4,924
6	TRIM28	37	88,549	9313400000	291040000	282	6,753	3,310
7	FSD1	17	55,819	624910000	24996000	49	6,558	2,489
8	WAPAL	19	132,94	344040000	4986100	40	6,266	5,266
9	NDUFS3	13	30,241	859670000	50569000	64	4,960	1,991
10	PNN	12	81,627	948840000	27907000	30	4,666	1,030
**THP-1**								
1	STIM1	20	77,422	1368800000	42776000	56	6,936	5,598
2	NDUFA5	6	13,459	443870000	88774000	31	5,845	4,450
3	NDUFS3	13	30,241	859670000	50569000	64	5,749	5,237
4	WAPAL	19	132,94	344040000	4986100	40	5,565	2,789
5	NR3C1	16	82,903	254870000	6216400	41	5,518	3,529
6	CDK11	24	89,437	1090800000	26605000	125	5,457	3,908
7	RPS9	19	22,591	4188400000	380760000	125	5,446	4,517
8	TRIM28	37	88,549	9313400000	291040000	282	5,312	1,667
9	NDUFS2	14	52,545	691600000	27664000	66	5,048	3,949
10	CKAP5	49	218,52	1061600000	10307000	157	4,805	6,160
⋮								
18	AMPD2	43	100,69	16639000000	415980000	308	3,338	3,708

Immunoprecipitation from HEK293, Jurkat and THP-1 whole cell lysates was performed using the anti-GR antibody clone 5E4. Differential protein abundance (fold change) compared to isotype control

(mouse IgG1) was calculated using two-sample Student's t test. NR3C1 encodes for the glucocorticoid receptor (GR). Legend: iBAQ, intensity-based absolute quantification; MW, molecular weight.

Supplementary Table S2: Mass spectrometric analyses of top enriched proteins immunoprecipitated from HEK293 whole cell lysates using different anti-GR antibodies

	Gene name	Unique peptides	MW [kDa]	Intensity	iBAQ	MS/MS count	fold change (log2)	p-value (-log10)
**HEK293: anti-GR (5E4) Lot#1**								
1	AMPD2	62	92,07	207880000000	5774600000	670	13,412	3,867
2	MYCBP2	126	510,08	31355000000	143170000	366	10,713	5,161
3	P4HB	36	57,116	48236000000	1378200000	265	10,605	3,319
4	SEC23IP	42	111,08	31193000000	779820000	192	10,254	4,021
5	CDK11B	4	89,437	27341000000	666840000	227	10,013	5,960
6	PNN	35	81,627	29871000000	878560000	233	9,801	4,272
7	CKAP5	81	225,49	34132000000	322000000	273	9,774	2,845
8	GLDC	53	112,73	32458000000	721290000	312	9,704	6,502
9	TRIM28	39	88,549	117530000000	3672800000	485	9,699	5,507
10	NDUFA5	10	13,459	17925000000	3585000000	107	9,646	4,402
⋮								
45	NR3C1	16	76,185	6895700000	197020000	80	6,752	3,906
**HEK293: anti-GR (5E4) Lot#2**								
1	AMPD2	62	92,07	207880000000	5774600000	670	13,360	3,857
2	CDK11B	4	89,437	27341000000	666840000	227	9,412	5,830
3	PNN	35	81,627	29871000000	878560000	233	9,015	4,178
4	FSD1	24	55,819	22212000000	888460000	232	8,679	5,830
5	TRIM28	39	88,549	117530000000	3672800000	485	8,259	5,297
6	NDUFA5	10	13,459	17925000000	3585000000	107	8,175	4,117
7	NDUFS2	21	52,545	18813000000	752520000	153	8,154	4,845
8	GLDC	53	112,73	32458000000	721290000	312	8,095	6,161
9	P4HB	36	57,116	48236000000	1378200000	265	7,476	2,727
10	PGAM5	21	32,004	10225000000	511270000	101	7,190	5,413
11	NR3C1	16	76,185	6895700000	197020000	80	7,112	3,893
**HEK293: anti-GR (G-5)**								
1	NR3C1	16	76,185	6895700000	197020000	80	7,404	2,941
2	PKM	25	57,936	5885200000	178340000	130	4,518	3,226
3	PRDX2	7	21,892	1241400000	95491000	12	4,075	4,352
4	CCDC65	2	57,296	800890000	28603000	2	4,065	2,311
5	ATP5A1	12	54,493	3270400000	116800000	52	3,777	2,108
6	RPS17	5	64,532	880420000	35217000	12	3,743	1,778
7	RPL27A	4	12,201	1556400000	259410000	38	3,606	2,752
8	RPS28	2	7,8409	236040000	78679000	5	3,586	3,953
9	RPL14	5	14,558	495580000	82596000	19	3,560	2,286
10	HSPA	2	71,027	664280000	17954000	23	3,028	3,043
**HEK293: anti-GR (pAb PA1)**								
1	YME1L1	20	75,981	8449900000	216670000	88	9,129	4,611
2	DBT	9	53,486	1846800000	65957000	28	7,617	4,983

3	ALDH18A1	4	87,088	12522000000	272230000	56	6,882	4,734
4	TLE4;TLE2	2	14,876	1669500000	278250000	30	6,404	4,945
5	DPY30	4	11,25	1774600000	443660000	25	6,030	3,316
6	ERH	4	12,259	1321900000	264380000	20	5,561	3,362
7	NR3C1	16	76,185	6895700000	197020000	80	5,214	2,968
8	NF1	8	319,37	225240000	1608900	13	4,606	4,142
9	HNRNPU	12	88,317	2857900000	84057000	54	4,600	3,177
10	THOC2	7	182,77	231160000	3082100	14	4,498	2,705

Immunoprecipitation from HEK293 whole cell lysates was performed using the anti-GR antibodies clones 5E4 (Lot#1, Lot#2) and G-5 as well as a polyclonal antibody (pAb PA1). Differential protein abundance (fold change) compared to isotype control (mouse IgG1, mouse IgG2b kappa and rabbit IgG, respectively) was calculated using two-sample Student's t test. NR3C1 encodes for the glucocorticoid receptor (GR). Legend: iBAQ, intensity-based absolute quantification; MW, molecular weight.

Supplementary Table S3: Mass spectrometric analysis of top depleted proteins by pre-incubation with APTEK-26 peptide before immunoprecipitation using the anti-GR (5E4) antibody

	Gene name	Unique peptides	MW [kDa]	Intensity	iBAQ	MS/MS count	fold change (log2)	p-value (-log10)
1	TRIM28	55	88,549	97946000000	3060800000	318	-13,174	5,310
2	ASNS	26	62,168	10486000000	374510000	98	-9,359	5,460
3	CKAP5	74	218,52	8121600000	78850000	182	-9,296	6,326
4	WAPAL	47	132,94	5794800000	83983000	155	-8,787	4,766
5	STIM1	37	77,422	6843400000	213860000	102	-8,715	4,887
6	NDUFS3	19	30,241	5091100000	299480000	57	-8,294	4,522
7	SEC13	11	35,54	2364800000	181910000	31	-8,138	5,676
8	SSR4	7	18,998	5623700000	803390000	27	-7,991	4,882
9	CDK11B	2	89,437	2811300000	68568000	50	-7,970	6,642
10	P4HB	25	57,116	2625700000	75020000	65	-7,860	4,578
⋮								
15	AMPD2	55	100,69	73870000000	1846800000	185	-7,202	2,410
⋮								
184	NR3C1	20	85,658	7084000000	172780000	103	-0,969	0,750

Immunoprecipitation from HEK293 whole cell lysates was performed using the anti-GR antibody clone 5E4 with and without pre-incubation with APTEK-26 peptide. Differential protein abundance (fold change) of samples with and without APTEK-26 peptide was calculated using two-sample Student's t test. NR3C1 encodes for the glucocorticoid receptor (GR). Legend: iBAQ, intensity-based absolute quantification; MW, molecular weight.

Figures

Supplementary Figure S1

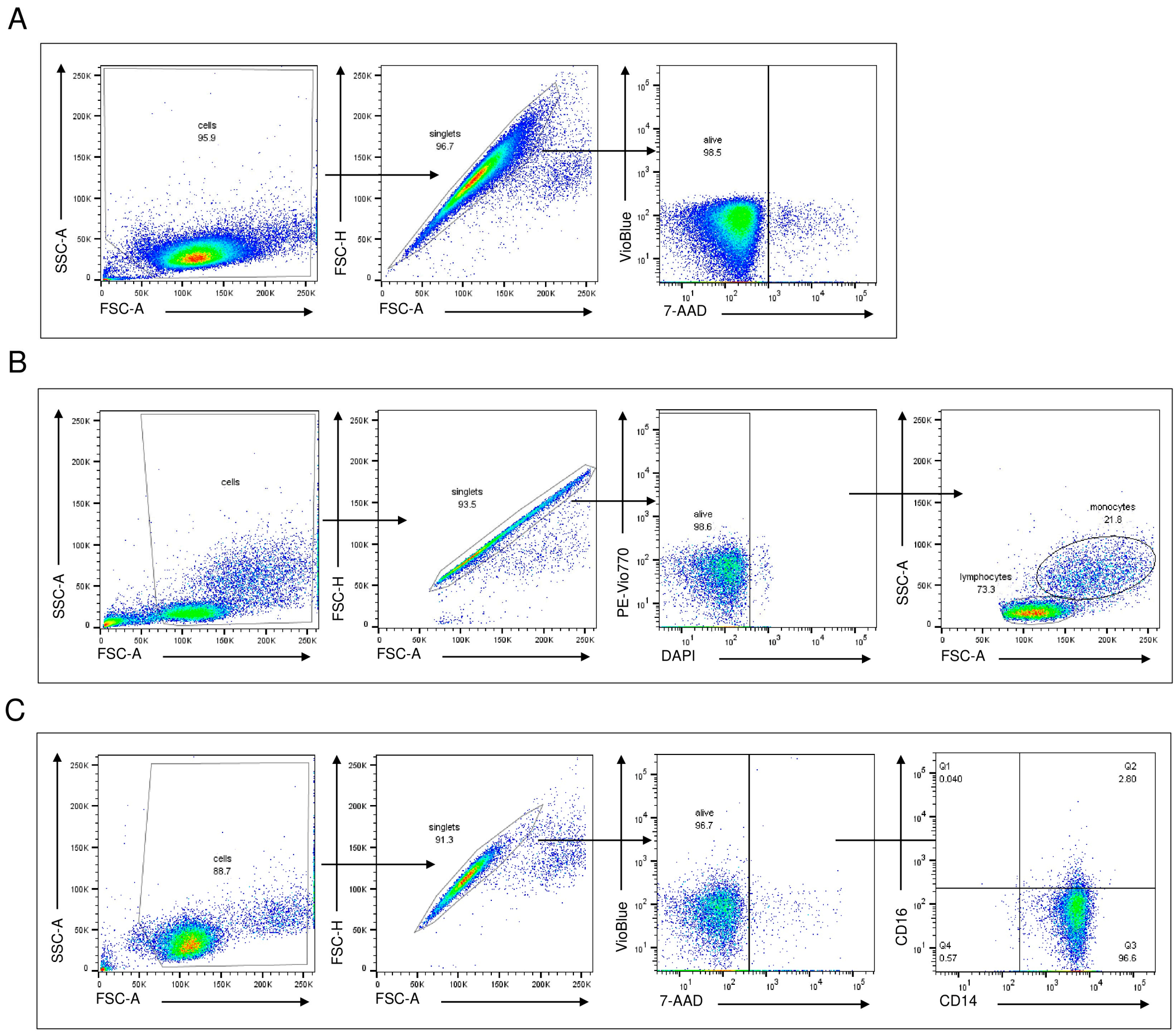

Supplementary Figure S1: Gating strategy. (A) Gating strategy. Cell lines were gated using a forward-scatter and side-scatter plot. Doublets were excluded according to the forward-scatter area and height pattern and 7-AAD was used to exclude dead cells. (B) PBMCs were gated using a forward-scatter and side-scatter plot. Doublets were excluded according to the forward-scatter area and height pattern and DAPI was used to exclude dead cells. A forward-scatter and side-scatter plot served to distinguish between the lymphocyte and monocyte populations. (C) CD14+ monocytes were sorted by magnetic cell separation. Cells were gated using a forward-scatter and side-scatter plot. Doublets were excluded according to the forward-scatter area and height pattern and 7-AAD was used to exclude dead cells. Surface staining of CD14 revealed purity of > 96%.

Supplementary Figure S2

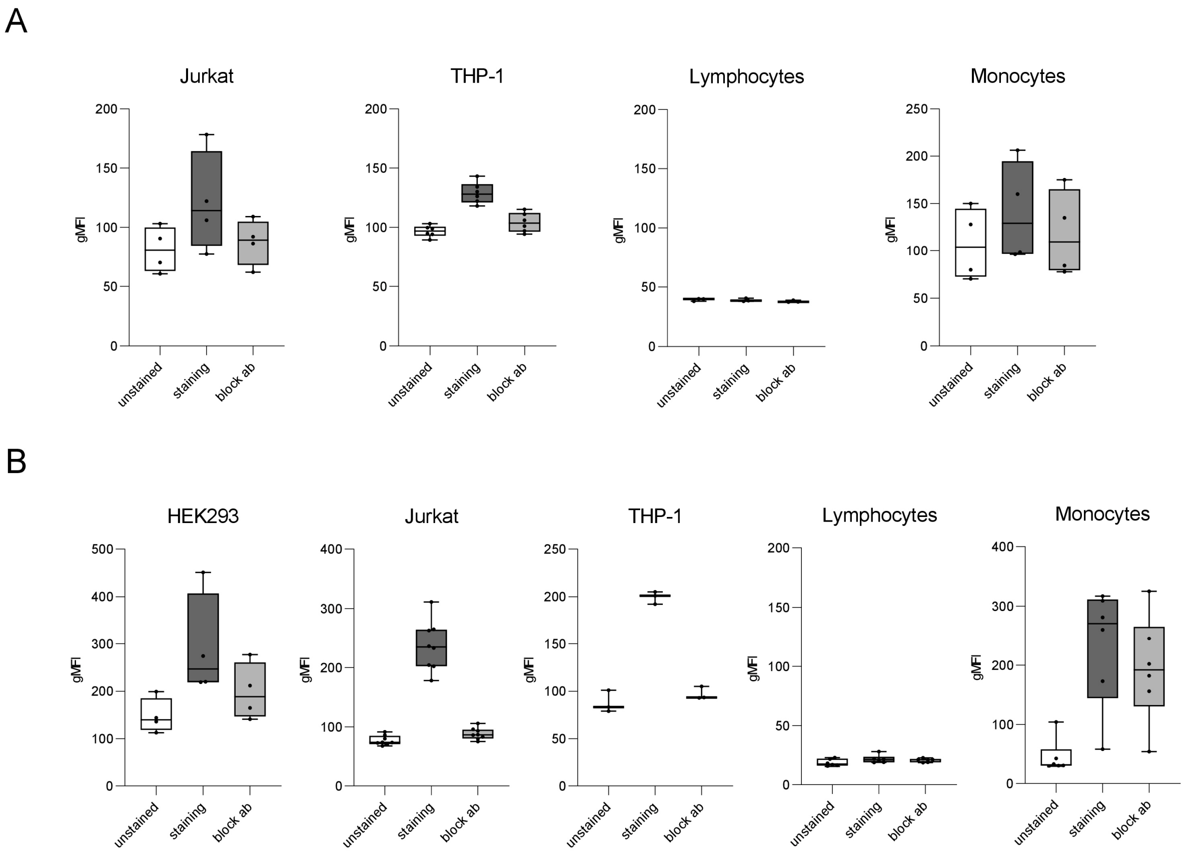

Supplementary Figure S2: Anti-GR surface staining. Multiple cell lines and primary human immune cells were analyzed for mGR expression by flow cytometry using the anti-GR antibody clones 5E4 (A) and G-5 (B). The staining was blocked successfully by ten-minute incubation with 100-fold (5E4) and 10-fold (G-5) excess unconjugated primary antibody (block ab) prior to the staining procedure. The gating strategy is displayed in *Supplementary Figure S1*. Staining intensities are depicted as geometric mean fluorescence intensity (gMFI). All boxplots show median, interquartile range, and minimum and maximum values, respectively.
